# Rhizosphere microbiome metagenomics in PGPR-mediated alleviation of combined stress from polypropylene microplastics and Cd in hybrid Pennisetum

**DOI:** 10.3389/fmicb.2025.1549043

**Published:** 2025-02-17

**Authors:** Si-Yu Zhao, Yue-Liang Meng, Zi-Han Yang, B. Larry Li, Yu-Ying Li, Hui Han, Ling Liu, Peng-Fei Duan, Zhao-Jin Chen

**Affiliations:** ^1^Collaborative Innovation Center of Water Security for Water Source Region of Mid-Route Project of South-North Water Diversion of Henan Province, Nanyang Normal University, Nanyang, China; ^2^Overseas Expertise Introduction Center for Discipline Innovation of Watershed Ecological Security in the Water Source Area of the Mid-line Project of South-to-North Water Diversion, Nanyang, China; ^3^College of Agriculture, Henan University of Science and Technology, Luoyang, China

**Keywords:** plant growth-promoting rhizobacteria, polypropylene (PP), hybrid Pennisetum, metagenomics, microbial function

## Abstract

The simultaneous presence of microplastics (MPs) and heavy metals in soil may result in heightened toxicity, causing more significant adverse effects on plant growth. Plant growth-promoting rhizobacteria (PGPR) have demonstrated significant capacities in alleviating the toxic stress caused by the combined pollution of heavy metals and other contaminants. However, research on the impacts and processes of PGPR in alleviating stress induced by the combined pollution from MPs and heavy metals is still insufficient. This study involved a pot experiment to evaluate the ability of PGPR to mitigate stress induced by the combined pollution from polypropylene microplastic (PP MPs) particles of different sizes (6.5 μm and 830 μm) and the heavy metal cadmium (Cd) in the bioenergy plant hybrid Pennisetum. Moreover, metagenomic analysis was used to examine the effects of PGPR on the rhizospheric microbial community and function. The cocontamination of PP and Cd affected the growth of the hybrid Pennisetum differently depending on the size of the MPs particles, with the aboveground and underground lengths of the 6.5 μm PP + Cd experimental group being smaller than those of the 830 μm PP + Cd group. The PGPRs (*Bacillus* sp. Y-35, *Bacillus* sp. Y-62, *Bacillus* sp. Y-S, and *Enterobacter* sp. Y-V) successfully alleviated the stress caused by the combined pollution of PP and Cd, resulting in increases of 8.24 and 42.21% in the plant height and dry weight, respectively. The metagenomic studies indicated that the cocontamination of PP and Cd, along with PGPR inoculation, altered the composition of the rhizospheric bacterial community, leading to changes in microbial diversity indices and the composition of dominant groups such as Pseudomonadota, Actinomycetota, and Acidobacteriota. The functional analysis revealed that the main functional groups involved glucose metabolism, energy metabolism, signal transduction, and nucleotide metabolism. The MPs particle size and different PGPR significantly affected functions such as the pentose phosphate pathway, benzoate degradation, and amide biosynthesis. This study provides essential data and scientific evidence on the ecotoxicological effects of simultaneous contamination by MPs and heavy metals, as well as insights into potential bioremediation methods.

## 1 Introduction

Microplastics (MPs), regarded as emerging contaminants, are plastic particles smaller than 5 mm (Lodeiro et al., 2019; Wang et al., 2020a). In recent years, the extensive utilization of microplastics in industry and daily activities has resulted in a considerable buildup of plastic trash in terrestrial ecosystems, including agricultural soils. This has raised particular concerns about the environmental impact of MPs (de Souza Machado et al., 2019; Huang et al., 2023; Nizzetto et al., 2016). MPs can infiltrate soils through several routes, including the application of biofertilizers (Ng et al., 2018). Organic fertilization, air deposition, and wastewater irrigation using plastic mulching sheets also cause MPs to enter soils (Weithmann et al., 2018). The presence of MPs in soils can modify the physical and chemical characteristics of the soil, impacting soil microorganisms, animal communities, and plants in various ways, posing a potential threat to agricultural ecosystems (Dong et al., 2020; Liu Y.-Q. et al., 2023). Modifications in water retention capacity and soil bulk density, aggregation, soil porosity, and microbial activity caused by MPs are fundamental factors that influence plant growth (Chen et al., 2020; Dong et al., 2021). Studies have indicated that microplastics are poisonous to hybrid Pennisetum and can hinder the growth of maize and water celery. These findings suggest that MPs in the ground can influence food safety. Therefore, the effects of MPs and nanoscale plastics (less than 100 nm in size) on agricultural ecosystems deserve more attention (Wang et al., 2020b).

Heavy metal contamination of soil has been identified as an increasingly serious global issue, exacerbated by human activities and industrial production, and poses a significant threat to the environment and human health (Naveed et al., 2020; Zhu et al., 2024). Cadmium (Cd) is one of the most common and toxic metallic pollutants in the environment (Talukder et al., 2023). According to the 2014 China Soil Pollution Report, Cd ranks first among metallic pollutants in farmland, exceeding the national soil environmental quality standards (Duan et al., 2024; Pinto-Poblete et al., 2022).

Studies have demonstrated that both MPs and heavy metals are persistent contaminants in soil and that their interactions can affect the toxicity and bioavailability of Cd in the soil–plant system (Huang et al., 2024). MPs play a crucial role in the transport of heavy metals in soil via processes such as physical adsorption and surface complexation. The combination of MPs and heavy metal pollution can alter the soil microbial community structure and rhizosphere metabolites, in turn affecting plant growth and development. Understanding how MPs particle size affects cadmium absorption by plants is important. Investigations on Cd absorption were conducted using polypropylene (PP) particles (5 μm, 10 μm) and polystyrene (PS) particles (50 nm, 100 nm) of two sizes (Zhao et al., 2023). The results suggested that PS measuring 100 nm enhances the absorption of Cd by maize. Another study revealed that Cd accumulation in rice can be decreased by 51 μm polylactic acid (PLA) particles. Research has demonstrated that MPs size affects the interaction between MPs and Cd, thereby influencing ecological consequences. Furthermore, smaller MPs might destroy bacterial cells, whereas larger MPs may facilitate more suitable environments (Colpaert et al., 2024). Although the role of plant microbiomes in the interactions between MPs and Cd has attracted much interest, the particular involvement of plant microbiomes in Cd absorption under the impact of MP particles of various sizes remains unresolved, as does the potential mechanisms by which MPs affect the soil microenvironment (An et al., 2024).

Plant growth-promoting rhizobacteria (PGPR) are beneficial bacteria typically found in the rhizosphere and on the root surface. They can increase or regulate plant growth and development through various mechanisms, such as nitrogen fixation, iron chelator production, biological nitrogen fixation, the activity of 1-aminocyclopropane-1-carboxylate deaminase (ACC deaminase), and the production of plant hormones (Zhang et al., 2024). Researchers have previously reported that the addition of PGPR can alter the forms of heavy metals in the soil, thereby reducing the migration of heavy metals into plant tissues. In previous studies, approaches to reduce the toxicity of MPs and heavy metals co-contaminating plants have been explored. The application of PGPR stimulates plant development and helps reduce the harmful effects of cocontamination (Liu Y. et al., 2023). PGPR can grow and survive under high concentrations of metals and actively plays a role in inducing many physiological and biochemical changes in plants. This, in turn, either directly or indirectly controls heavy metal absorption by plants and enhances their resistance to toxicity (Zhang et al., 2025). The activation of inorganic soil nutrients by PGPR is a crucial method by which these bacteria enhance plant development. Inoculation with PGPR enhances the solubility of vital nutrients, including nitrogen and phosphorus, in the soil, thus promoting plant development. This helps improve the nutrient content in the soil, which supports improved plant growth. Research has shown that inoculation with *Burkholderia* sp. D54 and *Bacillus* sp. TWD-2 improved the photosynthetic rate of rice plants and reduced heavy metal concentrations. Inoculation with the PGPRs *Enterobacter* sp. VY-1 and *Bacillus* sp. SL-413 under hydroponic conditions alleviated the stress of Cd + PE cocontamination on sorghum from our previous study, which was consistent with these findings (Liu Y. et al., 2023). These findings demonstrate that PGPR have beneficial effects across different environments.

Metagenomics, as a next-generation sequencing technology, allows the direct analysis of microbial DNA in the environment, providing insights into the genetic, functional, and ecological characteristics of microbial communities. The functional diversity of soil microorganisms may be thoroughly and precisely studied through the application of soil metagenomics (Meng et al., 2023). The bioenergy plant hybrid Pennisetum species, derived as a cross between American wheatgrass (*Elymus canadensis*) and bulrush (*Schoenoplectus*), is a perennial, tall, herbaceous plant. The hybrid Pennisetum exhibits strong stress resistance, wide adaptability, and high biomass yields. Moreover, recent studies have shown that hybrid Pennisetum demonstrates strong tolerance to Cd, moderate accumulation capacity, and the ability to extract significant amounts of cadmium from the soil (Gao et al., 2024). Additionally, hybrid Pennisetum has application value in anaerobic digestion for biogas production, direct combustion for power generation, pulp and paper production, and the manufacture of regenerated cellulose membranes (Jia et al., 2022). PGPR have been widely used to alleviate composite pollution caused by heavy metals, MPs, and pesticide residues, indicating their effectiveness in reducing plant stress and promoting plant growth (Pu et al., 2024). However, there is currently limited research on the use of PGPR to alleviate the cocontamination of MPs and heavy metals in hybrid Pennisetum. The exact mechanism by which PGPR exert their effects through the regulation of the rhizospheric microbiome remains unclear.

Therefore, this study attempted to answer the following two questions: (1) What is the role of PGPR in mitigating the combined effects of MPs particle size and Cd contamination? (2) How does PGPR alleviate the combined stress of PP + Cd pollution in the bioenergy plant hybrid Pennisetum by altering the rhizospheric microbial community and functions? In this study, the effects of PGPR isolated and screened in the laboratory on the growth of hybrid Pennisetum and Cd accumulation under composite pollution with polypropylene microplastic (PP MPs) particles of different sizes (6.5 μm and 830 μm) and Cd (10 mg kg^−1^) were analyzed via pot experiments. Additionally, metagenomic studies investigated how PGPR inoculation affects soil microbial communities and function. The findings offer basic information as well as scientific evidence for the ecotoxicological consequences of MPs-heavy metal pollution in agricultural systems and the possibility of bioremediation with bioenergy plants.

## 2 Materials and methods

### 2.1 Experimental materials

The bioenergy plant hybrid Pennisetum seeds used in the experiments were obtained from Muyang County Tuojing Horticultural Co., Ltd. The seeds chosen for the experiments were comparable in size, full-bodied, and free from surface damage. PP MPs particles with sizes of 6.5 μm and 830 μm were purchased from China Petroleum & Chemical Corporation (Sinopec). The test strains were selected from four strains with good growth-promoting characteristics that were previously screened in our laboratory: *Bacillus* sp. Y-35, *Bacillus* sp. Y-62, *Bacillus* sp. Y-S, and *Enterobacter* sp. Y-V. The screening studies revealed that all four strains generate iron carriers and indole-3-acetic acid (IAA), dissolve phosphorus, solubilize potassium, and exhibit good tolerance to Cd. On the west campus of Nanyang Normal University, Nanyang City, Henan Province, test soil was collected from the region around the pomegranate garden. The sampling depth was 0–20 cm for the surface soil. The basic physical and chemical characteristics of the soil were as follows: the soil was yellow-brown.; the organic matter content was 10.29 g kg^−1^; the cation exchange capacity was 16.8 cmol kg^−1^; the total phosphorus content was 1.90 g kg^−1^; the total nitrogen content was 1.31 g kg^−1^; and the pH was 6.93. No Cd was detected in the soil. The soil was air-dried naturally, reduced to a fine powder, and then passed through a 20-mesh screen for eventual usage once contaminants and plant wastes were eliminated.

### 2.2 Experimental methods

To reach a Cd ion concentration of 10 mg kg^−1^, the sieved soil for the pot treatment was first combined with CdSO₄·8H₂O. After thorough stirring to ensure an even distribution, the soil was air-dried for 1 month to fix the heavy metals. A net weight of 0.75 kg of the prepared soil was then placed into each pot for later use. Two PP MPs particle sizes (6.5 μm and 830 μm) with a PP concentration (*ω*) of 0.1% were used in the experimental design. Two treatment groups were set with PP + Cd composite pollution for each PP particle size (6.5 μm and 830 μm), and two treatment groups were set with PP + Cd composite pollution and inoculation of plant growth-promoting bacteria for each particle size. Ten treatment groups, with three replicates for each treatment, for a total of 30 plants. During the pot planting period, irrigation was used to maintain the soil moisture content at approximately 75% (Supplementary Figure S1). The specific experimental design is shown in Table 1.

**Table 1:** Design of the experiments.

Experimental grouping	PP MPs particle/μm	PGPR
6.5 μmPP + Cd	6.5	No
6.5 μmPP + Cd + Y-35	6.5	*Bacillus* sp. Y-35
6.5 μmPP + Cd + Y-62	6.5	*Bacillus* sp. Y-62
6.5 μmPP + Cd + Y-S	6.5	*Bacillus* sp. Y-S
6.5 μmPP + Cd + Y-V	6.5	*Enterobacter* sp. Y-V
830 μmPP + Cd	830	No
830 μmPP + Cd + Y-35	830	*Bacillus* sp. Y-35
830 μmPP + Cd + Y-62	830	*Bacillus* sp. Y-62
830 μmPP + Cd + Y-S	830	*Bacillus* sp. Y-S
830 μmPP + Cd + Y-V	830	*Enterobacter* sp. Y-V

### 2.3 Inoculation of the tested strains

The chosen strains (strains demonstrating strong growth-promoting properties) were inoculated into liquid LB media and then cultured at 28°C under shaking for 12 to 24 h. Some of the culture mixture was then piped into a 50 mL centrifuge tube and spun for 20 min at 4,800 r min^−1^ to harvest the bacterial cells. After several washes with sterile deionized water, the cells were resuspended at 1 × 10^8^ CFU mL^−1^. When the seedlings were 30, 45, or 60 days old, the tested strains were inoculated by applying 10 mL of the bacterial suspension to the roots of each hybrid Pennisetum plant in each pot.

### 2.4 Pot treatment

After 80 days of growth, the hybrid Pennisetum plants were harvested along with the rhizospheric soil samples. After the plants were carefully removed from their pots, the soil from the roots was gently shaken to collect the rhizospheric dirt. The roots were then submerged in a 0.01 mol L^−1^ EDTA-2Na buffer solution for 20 min and rinsed with pure water. Afterward, the plants were dried in an oven at 100°C for 24 h in envelopes. The dry weights of the aboveground and subterranean sections of the plants were subsequently recorded. The aboveground and underground parts were separately ground to powder and digested, and ICP-OES was used to assess the Cd concentration in the plant tissues. During the analysis, certified soil (GBW 07428) and plant (GBW 10015) reference materials were added for quality control. After being naturally air-dried, the soil was crushed and passed through 1 mm and 1.5 mm sieves. The physicochemical properties are then determined, with the soil pH measured via a pH meter. Using the extraction-molybdenum-antimony anti-spectrophotometric approach, the amount of accessible phosphorus was ascertained; the available potassium (AK) concentration was determined via flame photometry; and the alkaline hydrolyzable nitrogen content was determined via the alkaline hydrolyzation diffusion technique.

### 2.5 Metabolomic analysis

Metagenomic sequencing was carried out using the Illumina NovaSeq sequencing platform supplied by Shanghai Majorbio Biotechnology Co., Ltd. (Duan et al., 2024). The process was as follows: one end of the library molecules was complementary to the primer bases. After amplification, the template information was fixed onto a chip. The other end of the molecule randomly bound to a nearby primer, forming a “bridge.” Through another round of amplification, DNA clusters were formed, and further amplification was performed to convert them to single-stranded DNA. Modified DNA polymerase and dNTPs labeled with four different fluorescent tags were added. In each cycle, one nucleotide base was synthesized. A laser was used to scan the reaction plate to determine the type of nucleotide in each template sequence included. The “terminator group” and “fluorescent group” were cleaved, and the second nucleotide was incorporated. The fluorescent signals were collected in each cycle, and the raw sequence data were obtained. The resulting template DNA sequences were subjected to trimming, filtering, and decontamination to retain only high-quality paired-end reads and single-end reads. MEGAHIT v1.1.2 was used to combine the best sequences acquired (Li et al., 2016). The last assembly consisted of contigs of at least 300 bp in size. The contigs from the assembly results were subjected to gene open reading frame (ORF) prediction using Prodigal software. The genes whose nucleotide length was greater than 100 bp were selected and converted to amino acid sequences. The predicted gene sequences from all the samples were clustered using CD-HIT v4.6.1 software (with 95% identity and 90% coverage used as thresholds). The longest gene in each cluster was selected as the representative sequence to construct a nonredundant gene set. Finally, the high-quality readings of every sample were aligned against the nonredundant gene set (with a 95% identity criterion) via the SOA Paligner program. Then, the abundance of genes in the related samples was computed. The obtained genes were annotated and classified at the species and functional levels, including species abundance annotation using the NR (nonredundant) database, as well as functional annotations using databases such as eggNOG and KEGG.

### 2.6 Data analysis

All the data were obtained from three biological replicates; therefore, SPSS 23.0 was used for the statistical analysis. *T*-tests and one-way analysis of variance (ANOVA) were used for significance testing; the significance threshold was set at *p* < 0.05.

## 3 Results

### 3.1 Effects of the different treatments on the growth of the hybrid Pennisetum plants

**Figure 1:**
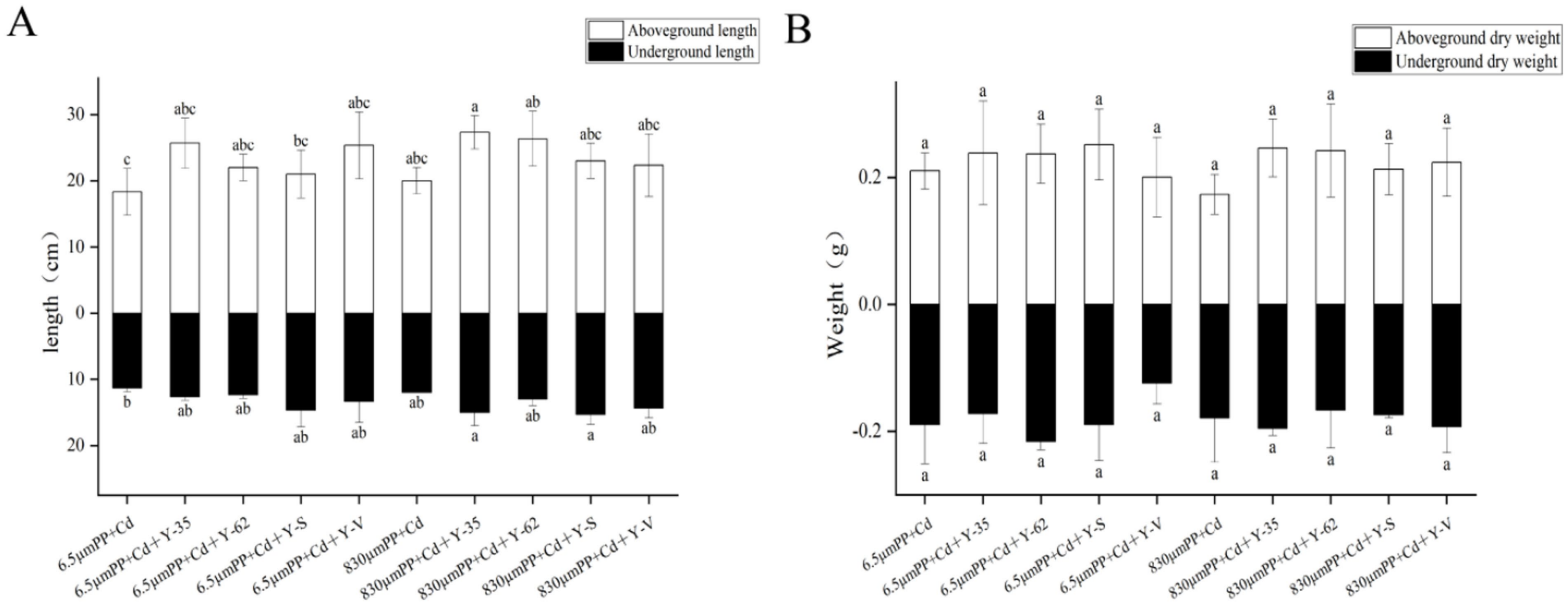
Length **(A)** and dry weight **(B)** of the hybrid Pennisetum plants under the different treatments. Different letters indicate significant differences between the treatments (*p* < 0.05).

As shown in Figure 1A, the lengths of both the aboveground and underground parts of the plants treated with 6.5 μm PP + Cd were lower than those of the plants treated with 830 μm PP + Cd, with reductions of 9.09 and 5.88%, respectively. In contrast to those in the 6.5 μm PP + Cd treatment group, the lengths of both the aboveground and underground parts in the PGPR treatment group significantly increased, ranging from 14.55 to 40.00% for the aboveground parts and 8.24 to 29.41% for the underground parts. Compared with those in the 830 μm PP + Cd-treated group, the lengths of the aboveground and underground parts in the PGPR treatment group increased by 11.67 to 36.67% and 8.33 to 27.78%, respectively. Among the different PGPR strains, *Bacillus* sp. Y-35 had the strongest growth-promoting effect on the aboveground parts, whereas *Bacillus* sp. Y-S led to the most significant growth in the underground parts.

As shown in Figure 1B, the effects of combined PP + Cd contamination and PGPR inoculation on the dry weight of hybrid Pennisetum were similar to those on length. Compared with that in the 6.5 μm PP + Cd treatment group, the aboveground dry weight decreased by only 4.54% in the 6.5 μm PP + Cd + Y-V treatment group, whereas the aboveground dry weight in the other inoculated treatment groups increased by 12.91 to 19.75%. Compared with the 830 μm PP + Cd treatment, the inoculated treatment groups resulted in an increase of 22.86 to 42.21% in the aboveground dry weight of the plants.

The cocontamination of PP and Cd affected the growth of the hybrid Pennisetum plants differently depending on the size of the MP particles. PGPR effectively alleviate the stress caused by the combined PP + Cd pollution, increasing the length and dry weight of the hybrid Pennisetum plants.

### 3.2 Effects of the treatments on the Cd concentration and accumulation in the hybrid Pennisetum plants

**Figure 2:**
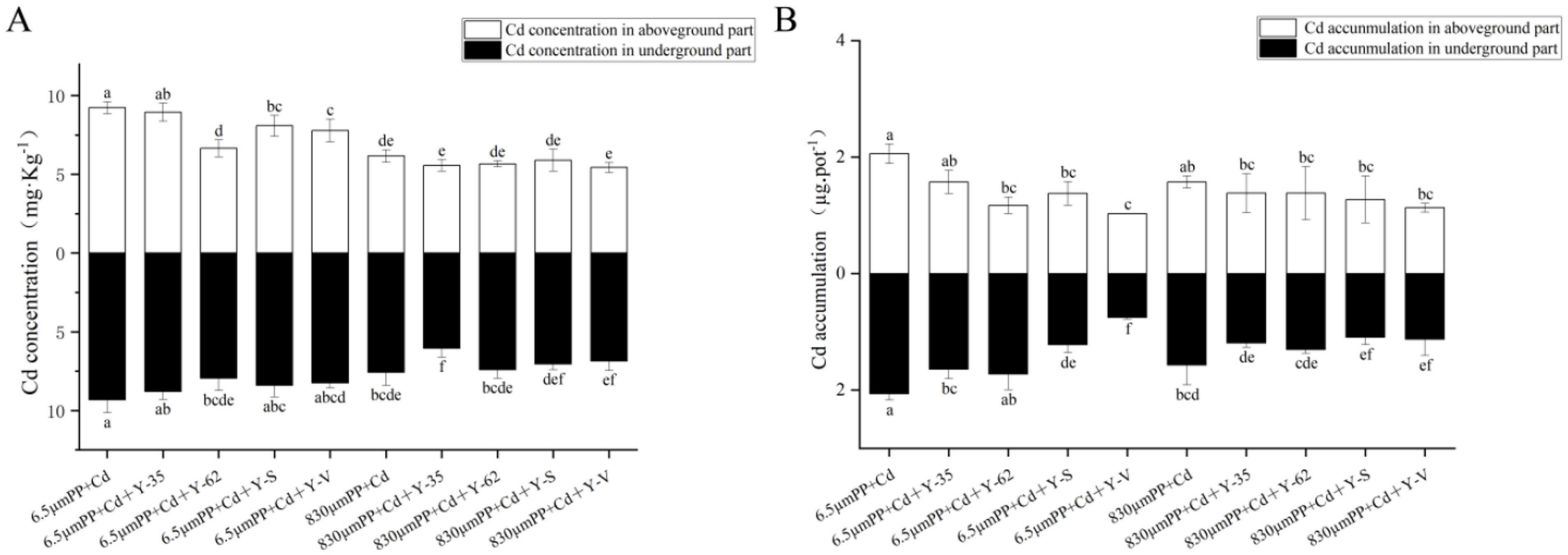
Cd concentration **(A)** and accumulation **(B)** in the hybrid Pennisetum plants under the different treatments. Different letters indicate significant differences between the treatments (*p* < 0.05).

The aboveground and underground Cd concentrations in the plants treated with 830 μm PP + Cd were 33.21% lower than those in the plants treated with 6.5 μm PP + Cd. Under the 6.5 μm PP + Cd treatment the use of PGPR lowered the Cd concentrations in the hybrid Pennisetum aboveground parts, ranging from 3.07 to 27.98%. Among the PGPR strains examined, *Bacillus* sp. Y-62 had the most notable effect on lowering Cd accumulation. The Cd concentration in the underground parts of the hybrid Pennisetum plants decreased by 5.72 to 14.67% after PGPR inoculation under the 6.5 μm PP + Cd treatment. Unlike the 830 μm PP + Cd treatment, PGPR inoculation led to a reduction in the Cd content in both the above- and underground parts, with decreases ranging from 4.32 to 11.89% in the aboveground tissues and from 2.41 to 9.45% in the underground tissues (Figure 2A).

As shown in Figure 2B, the accumulation of Cd in the above- and underground parts of the plants treated with 830 μm PP + Cd was 23.61 and 23.94%, respectively, lower than that in the plants treated with 6.5 μm PP + Cd. Under the 6.5 μm PP + Cd treatment, inoculation with PGPR resulted in a reduction in Cd accumulation in the aboveground and underground parts of hybrid Pennisetum, with decreases ranging from 13.59 to 74.78% in the aboveground parts and 16.41 to 63.41% in the underground parts. Among the PGPR strains, *Enterobacter* sp. Y-V had the most significant effect on reducing Cd accumulation. Compared with the 830 μm PP + Cd treatment, inoculation with PGPR significantly reduced Cd accumulation in the aboveground and underground parts of the hybrid Pennisetum plants, with decreases ranging from 12.16 to 28.02% in the aboveground parts and from 16.73 to 30.34% in the underground parts.

### 3.3 Effects of the different treatments on the bacterial community composition and diversity

The Venn diagram in Supplementary Figure S2 represents the composition of the bacterial microbiota across different groups, showing that a total of 429 bacterial genera were present in the samples. Among the 6.5-μm PP treatments, the 6.5-μm PP + Cd + Y-62 and 6.5-μm PP + Cd treatments presented the greatest number of unique bacterial genera, with 23 and 12 genera, respectively. Similarly, among the 830 μm PP treatments, the 830 μm PP + Cd + Y-V and 830 μm PP + Cd + Y-62 treatments presented the greatest number of unique bacterial genera, with 29 and 20 genera, respectively.

On the basis of gene-based taxonomic annotation and alignment with the NR database, species and abundance information at various taxonomic levels for each sample were obtained. A total of 30 soil samples from the rhizosphere of the hybrid Pennisetum plants were collected from 10 different groups. Through NR-based species annotation, 231 phyla and 5,217 genera were identified.

As shown in Supplementary Figure S3A, Pseudomonadota dominated the microbiology, contributing between 21.80 to 42.57% of all the bacteria, followed by Actinomycetota (19.52 to 27.26%), Acidobacteriota (8.80 to 12.34%), and Nitrososphaerota (6.69 to 10.72%). These four phyla together accounted for 56.81 to 92.89% of all the sequences. Among them, Pseudomonadota was predominant in the 6.5 μm PP + Cd + Y-62 treatment, with an abundance of 30.60%.

As shown in Supplementary Figure S3B, the total abundances of *Luteitalea* (2.82 to 4.34%), *Sphingomonas* (2.45 to 4.24%), *Rubrivax* (2.04 to 3.15%), and *Gaiella* (1.64 to 2.72%) accounted for 8.95 to 14.45% of all sequences. The above results indicate that different particle sizes of MPs and different PGPR alter the bacterial community composition in the rhizosphere soil of hybrid Pennisetum under the cocontamination of PP and Cd.

**Figure 3:**
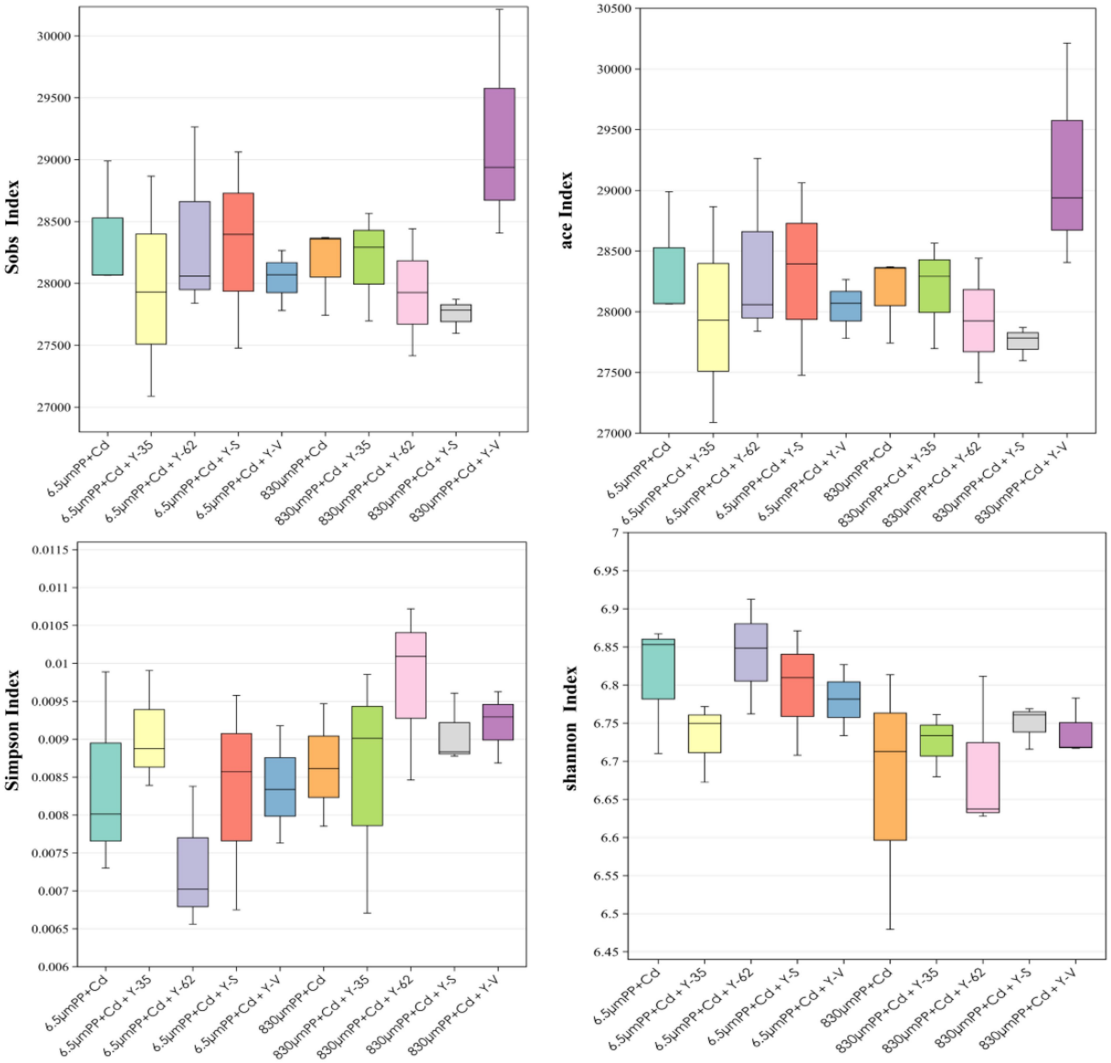
Rhizosphere microbial diversity indices of the soil of the hybrid Pennisetum plants under the different treatments.

As shown in Figure 3, under the cocontamination of PP + Cd, with increasing MPs particle size, the ACE index, Sobs index, and Simpson index all decreased. This suggests that, under cocontamination conditions, an increase in particle size reduces community diversity and abundance, resulting in changes in the structure of the bacterial population. In the 6.5 μm PP + Cd treatment, inoculation with *Bacillus* sp. Y-62 resulted in increases in the ACE index, Sobs index, and Shannon index, indicating increased community richness and diversity. In contrast, inoculation with *Bacillus* sp. Y-35 led to an increase in the Simpson index, suggesting a shift toward greater evenness in the bacterial community. The ACE index and Sobs index increased in the 830 μm PP + Cd treatment group after *Enterobacter* sp. Y-V inoculation, whereas the Shannon index increased upon inoculation with *Bacillus* sp. Y-S. Inoculation with PGPR altered the soil microbial community and diversity.

### 3.4 LEfSe analysis

**Figure 4:**
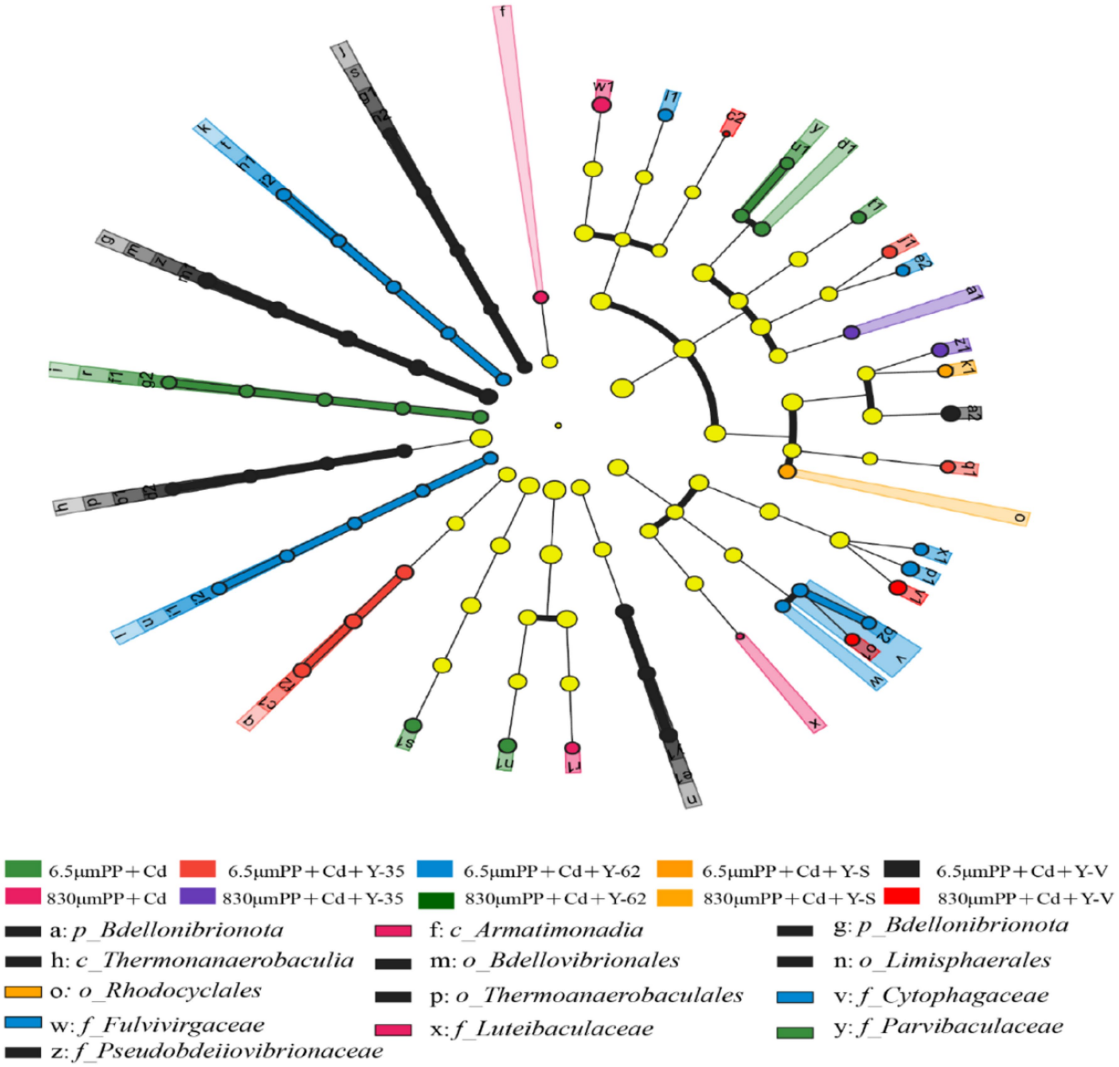
Differentially abundant bacteria between the different samples revealed by linear discriminant analysis effect size (LEfSe) analysis.

The variations in the bacterial community composition of the rhizospheric soil across the samples under the various treatment levels were investigated via linear discriminant analysis (LDA) effect size (LEfSe) software (Figure 4). The results revealed that under the condition of linear discriminant analysis (LDA) >2, 37 bacterial taxa in the rhizosphere soil differed between the treatments. In the 6.5 μm PP + Cd treatment group, the significantly differentially abundant taxa were Hyalangium, Blastococcus, and Parvibaculaceae. In the 6.5 μm PP + Cd + Y-35 treatment group, significant differences were observed in the abundances of Altererythrobacter and Gallionella. In the 6.5 μm PP + Cd + Y-62 treatment group, the significantly differentially abundant genera were Flavisolibacter, Cytophagaceae, Azotobacter, Rhodocytophaga, Fulvivirgaceae, Paraflavisolibacter, and Tsuneonella. In the 6.5 μm PP + Cd + Y-V treatment group, the significantly differentially abundant genera were Bdellovibrionota, Ramlibacter, Bdellovibrionia, and Thermoanaerobaculia. In the 830 μm PP + Cd + Y-35 treatment group, the significantly differentially abundant genera were Luteibaculaceaec, Humibacterg, Luteibacterp, and Armatimonadia. Rhodocyclales and Aquabacterium abundances were significantly different in the 830 μm PP + Cd + Y-S treatment group; Lacibacter, Chryseosolibacter, and Rodentibacter abundances were significantly different in the 830 μm PP + Cd + Y-V treatment group.

### 3.5 Metagenomic analysis of hybrid Pennisetum

#### 3.5.1 KEGG annotation analysis of the hybrid Pennisetum plants under the different treatments

**Figure 5:**
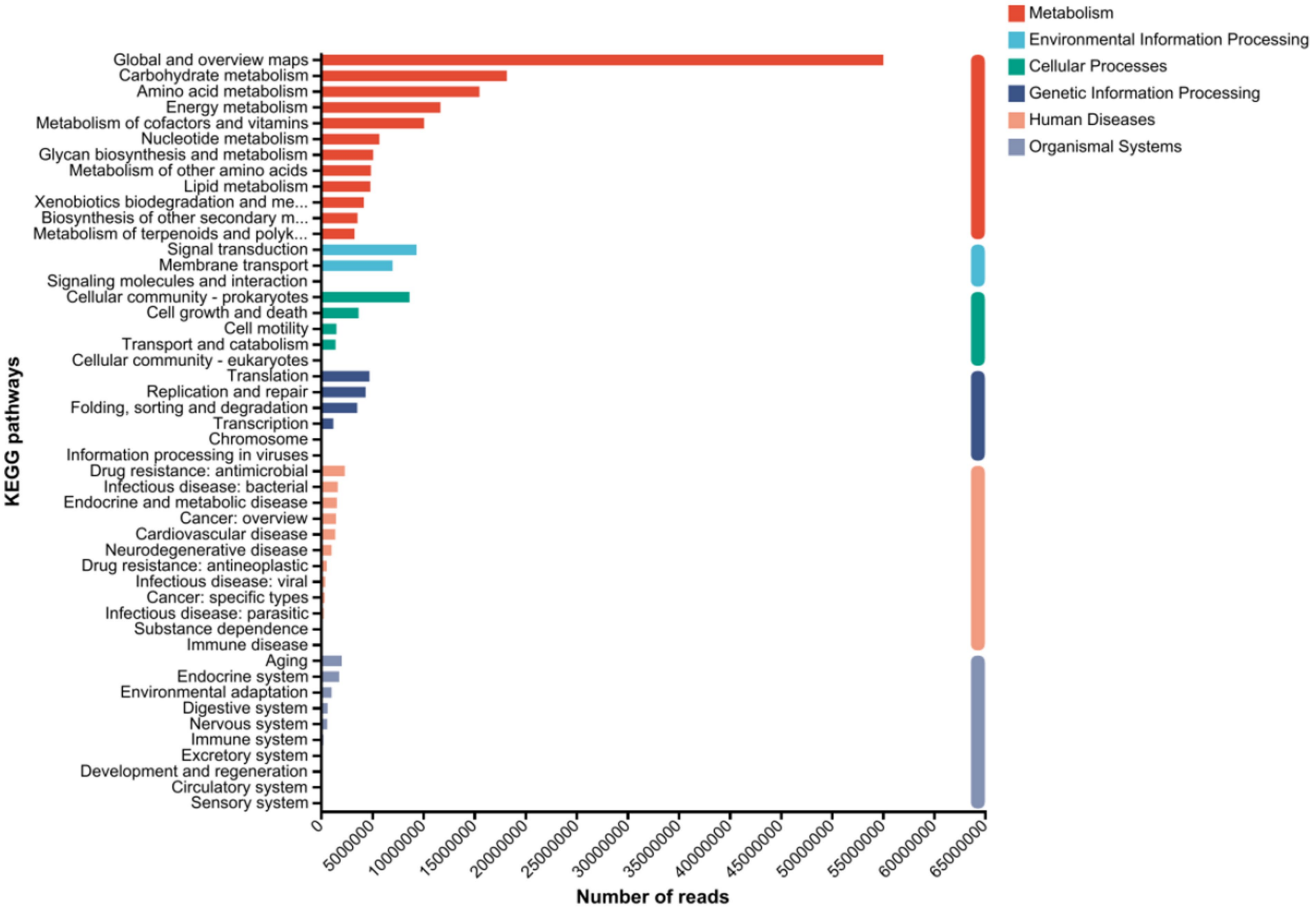
KEGG metabolic pathway annotation map of the hybrid Pennisetum plants under the different treatments.

The NR genes were annotated in the KEGG database, as shown in Figure 5. From the DNA sequences of 30 samples, 100,012 NR genes in total were assigned to 37 pathways spanning the six main KEGG categories: metabolism, environmental information processing, cellular processes, genetic information processing, human diseases, and organismal systems. Among the first-level KEGG metabolic pathways, the root-associated microorganisms of hybrid Pennisetum presented the highest abundance of metabolic pathways, making them the most dominant group. The abundance of metabolic pathways in the organismal system was the lowest, followed by those associated with environmental information processing, genetic information processing, and human diseases. The most representative KEGG secondary pathways were global and overview maps. Among the KEGG metabolic pathways at the secondary level, the most frequently annotated metabolic pathways included carbohydrate metabolism, amino acid metabolism, energy metabolism, signal transduction, cofactor and vitamin metabolism, and nucleotide metabolism. At the secondary level of the environmental information processing pathways, signal transduction and membrane transport were the most frequently annotated metabolic pathways. At the secondary level of the cellular process pathways, the most frequently annotated pathways were signal molecules and interactions with cellular communities-prokaryotes, as well as cell growth and death. At the secondary level of the genetic information processing pathways, translation, folding, and degradation were the most frequently annotated pathways. At the secondary level of the human disease pathways, drug resistance, antimicrobial and infectious diseases, and bacterial diseases were the most frequently annotated pathways. The KEGG database annotation results for the hybrid Pennisetum plants indicate that they contain relatively high abundances of carbohydrates and proteins. Therefore, genes related to carbohydrate metabolism and amino acid metabolism also presented relatively high abundances.

#### 3.5.2 COG functional annotation of the rhizosphere microorganisms of hybrid Pennisetum under the different treatments

**Figure 6:**
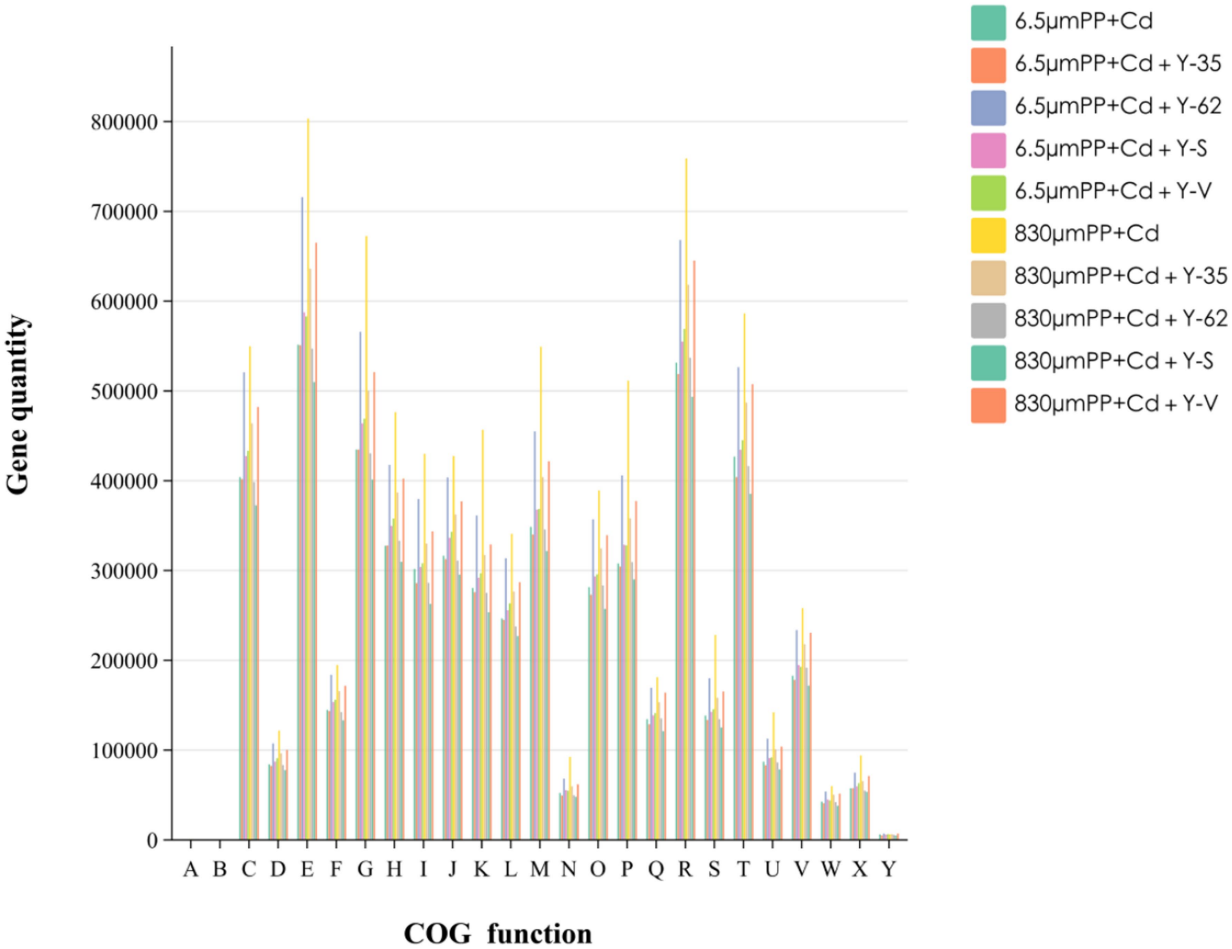
Gene sequence EggNOG database annotation statistics map of the hybrid Pennisetum plants under the different treatments. A: RNA processing and modification. B: Chromatin structure and dynamics. C: Energy production and conversion. D: Cell cycle control, cell division, chromosome partitioning. E: Amino acid transport and metabolism. F: Nucleotide transport and metabolism. G: Carbohydrate transport and metabolism. H: Coenzyme transport and metabolism. I: Lipid transport and metabolism. J: Translation, ribosomal structure and biogenesis. K: Transcription. L: Replication, recombination and repair. M: Cell wall/membrane/envelope biogenesis. N: Cell motility. O: Posttranslational modification, protein turnover, chaperones. P: Inorganic ion transport and metabolism. Q: Secondary metabolite biosynthesis, transport and catabolism. R: General function prediction only. S: Function unknown. T: Signal transduction mechanisms. U: Intracellular trafficking, secretion, and vesicular transport. V: Defense mechanisms. W: Extracellular structures. X: Mobilome: prophages, transposons. Y: Cytoskeleton.

After comparison with the EggNOG database, COG functional annotation was performed on the root-soil bacteria of the hybrid Pennisetum plants in the different treatment groups. The COG database contains annotations for four broad categories, which are further subdivided into 25 functional groupings, as shown in Figure 6. Under different treatments, the metabolic activities of the microorganisms significantly changed. The total abundance of metabolic pathways in the 8 sample groups was the highest, accounting for 47.87% of the total abundance, whereas those of information storage and processing pathways were the lowest, accounting for 14.97% of the total abundance. The key routes included amino acid transport and metabolism, Carbohydrate transport and metabolism, and cell wall/membrane/envelope biogenesis. Among the different treatment groups, the microbial activity in hybrid Pennisetum was related mainly to carbohydrate and amino acid metabolism. This is because cellular metabolism primarily uses carbon and nitrogen sources as raw materials, and their consumption promotes microbial absorption of nutrients and regulates related metabolic activities. Among them, the top three functional genes in terms of relative abundance were amino acid transport and metabolism, carbohydrate transport and metabolism, and signal transduction mechanisms, whereas chromatin structure and dynamics had the lowest relative abundances. In the composite pollution groups with the two different particle sizes, contamination with larger MPs particles (830 μm) + Cd led to a decrease in the relative abundance of chromatin structure and dynamics, whereas the relative abundances of other functional genes increased. The abundances of the three primary functional categories—amino acid transport and metabolism, carbohydrate transport and metabolism, and signal transduction mechanisms—increased by 45.64, 54.66, and 37.36%, respectively, under the 830 μm PP + Cd treatment. These findings suggest that the impact of different MPs particle sizes on function varies. Among the different treatment groups, the 830 μm PP + Cd treatment group presented the greatest number of genes in all 25 functional groups. On the other hand, the 6.5 μm PP + Cd + Y-35 and 830 μm PP + Cd + Y-S treatment groups presented the lowest number of genes across the 25 functional groups.

### 3.6 Differences in microbial function in the rhizospheric soil of hybrid Pennisetum

**Figure 7:**
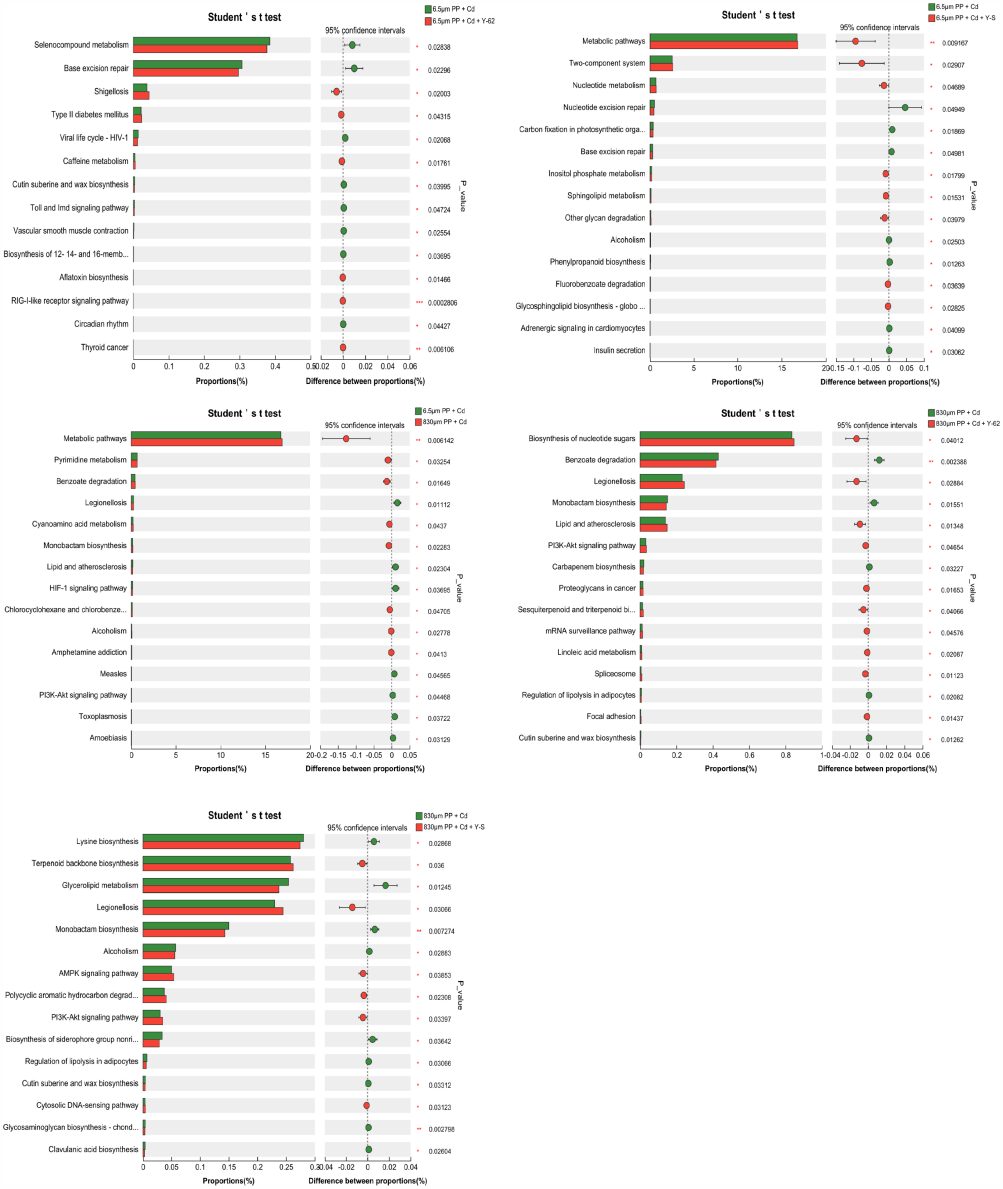
Variations in KEGG functional groupings among the hybrid Pennisetum rhizosphere bacteria.

To identify very distinct functions and metabolic paths between the groups, Fisher’s exact test was used to investigate the intergroup variances in KEGG functional metabolic paths across the treatments with varying particle sizes and PGPR inclusion. Additionally, the effect sizes of different functions were evaluated. As shown in Figure 7, when the MPs particle size and the added PGPR differed, significant differences in microbial metabolism, including pathways such as the pentose phosphate pathway, were observed between the different treatments. The differences between the treatments were consistent, but the concentrations varied. When the pairs of treatment groups were compared, significant differences were found in up to 15 groups. Compared with both the 6.5 μm PP + Cd + Y-S group and the 6.5 μm PP + Cd group, the 6.5 μm PP + Cd group presented significant differences in metabolic pathways. Significant differences in RIG-I-like receptor signaling and thyroid cancer pathways were detected between the 6.5 μm PP + Cd group and the 6.5 μm PP + Cd + Y-62 group. A significant difference in the degradation of benzoates was observed between the 830 μm PP + Cd group and the 830 μm PP + Cd + Y-62 group. The significantly different pathway between the 830 μm PP + Cd group and the 830 μm PP + Cd + Y-S group was the glycosaminoglycan biosynthesis. The above results indicate that under PP MPs (6.5 μm and 830 μm) and Cd cocontamination, the function of soil bacteria in the rhizosphere of the hybrid Pennisetum was strongly impacted by the addition of PGPR, with the size of the PP MPs (6.5 μm and 830 μm) particles and different PGPR playing key roles.

## 4 Discussion

### 4.1 PGPR alleviate the growth stress caused by PP + Cd cocontamination in hybrid Pennisetum

Under microbial and heavy metal contamination, PGPR can directly or indirectly increase plant growth, alleviate stress caused by environmental stressors, promote nutrient absorption by crops, and play crucial roles in maintaining soil ecological health and heavy metal accumulation (Liu et al., 2024). The four bacterial strains employed in this research were obtained from soil contaminated with heavy metals and MPs. The identification of these strains revealed that they were *Enterobacter* and *Bacillus* species, which are well-known and widely reported as PGPR strains (Dobrzyński et al., 2022). An investigation of the plant growth-promoting properties of these strains revealed that they can generate IAA, dissolve phosphorous, release potassium, and resist Cd toxicity. This study revealed that *Bacillus* and *Enterobacter* species, which have IAA production capabilities, play important roles in promoting root cell division and elongation. They also help alleviate heavy metal stress in plants and increase the potential for plant remediation. IAA plays a crucial role in promoting root cell division and elongation (de-Bashan et al., 2012). Iron-chelating bacteria and nitrogen-fixing bacteria can increase the nutrient utilization efficiency of plants (Tiwari et al., 2016).

Our findings suggest that PGPR inoculation could be an efficient alternative biological method for bioremediation. In this study, compared with the noninoculated control group, inoculation with PGPR under the PP + Cd composite pollution conditions significantly increased the length of the aboveground parts of the hybrid Pennisetum plants by 14.55 to 40.00% and 11.67 to 36.67%, respectively. Among them, the most significant increase in the length of the aboveground parts was observed in the plants inoculated with the *Bacillus* sp. Y-35 strain. The most notable increase in dry weight was found in the plants inoculated with *Bacillus* sp. Y-35, with the aboveground dry weight of the plants in the 830 μm PP + Cd treatment group increasing from 22.86 to 42.21%. The addition of PGPR effectively alleviated the stress of the PP + Cd cocontamination in the hybrid Pennisetum plants, promoting plant growth and development (Liu Y.-Q. et al., 2023, 2024; Pu et al., 2024; Zhang et al., 2024, 2025).

Previous reports have shown that the addition of PGPR significantly inhibits metal toxicity (Poppeliers et al., 2023). This phenomenon may be attributed to changes in the mechanisms by which PGPR mediate the regulation of metals, such as by reducing metal uptake into root tissues and altering the forms of metals during transport (Wang et al., 2022). These results indicate that inoculation with PGPR can improve plant stability and enhance the fixation of metals in the soil, thereby reducing the threat posed by metals to living organisms and the environment and promoting plant growth. This is consistent with the findings of the present study (Zhang et al., 2024, 2025). Studies have shown that PGPR regulate plant resistance by stimulating metal tolerance mechanisms, thereby promoting plant growth and yield (Islam et al., 2014; Mendoza-Cózatl et al., 2011; Sathyapriya et al., 2012).

In this study, after the bacterial inoculation, the Cd level in the aboveground and underground parts of the hybrid Pennisetum plants in the treatment group decreased to differing degrees. PGPR also helped ameliorate the plant stress resulting from the MP and heavy metal combined pollution by lowering the accumulation of Cd in the hybrid Pennisetum plants. This investigation revealed that the strains utilized generated IAA, siderophores, and soluble K. The results of the pot experiments revealed that under combined PP + Cd contamination conditions, these strains effectively alleviated the reduction in nutrients caused by PP MPs and heavy metals and promoted plant growth. These PGPR are excellent soil remediation agents capable of precipitating or adsorbing heavy metals in the soil, thereby significantly alleviating the toxicity of combined heavy metal and MP contamination in hybrid Pennisetum (Liu et al., 2024; Pu et al., 2024).

### 4.2 Effects of PGPR on the rhizospheric microbial community composition and function of hybrid Pennisetum plants

Inoculating PGPR into the rhizosphere not only affects plants but also alters the microbial population in the rhizospheric soil (Kong et al., 2022). The species composition and changes in the microbial community of the plant rhizosphere are important indicators of soil health and plant growth adaptability, as well as key factors that determine plant health and productivity. Many elements affect the makeup of soil microbial populations, including soil type, soil pollutants, and plant species.

In terms of environmental remediation, many studies have shown that PGPR can efficiently change the soil bacterial community composition and function and reduce the effects of stress generated by heavy metal and MPs cocontamination, thus increasing plant growth and increasing remediation efficiency (Nasuelli et al., 2023). This investigation revealed that under the PP + Cd cocontamination treatments with various particle sizes and inoculation techniques, the rhizobacterial community composition of the hybrid Pennisetum plants varied greatly. Inoculation with PGPR under the PP + Cd cocontamination treatment changed the bacterial community composition by increasing its variety and abundance. Habibollahi et al. (2017) reported that many bacteria play crucial roles in plant growth. In soils containing heavy metals, the main phyla include Proteobacteria, Firmicutes, Acidobacteria, and Actinobacteria, and they can alleviate the toxicity of heavy metals in plants, playing a key role in mitigating the harmful effects of heavy metal pollution. The present study revealed that the soil subjected to the PP + Cd cocontamination and bacterial inoculation also contained these dominant bacterial populations. These bacteria exhibit high adaptability and play crucial roles in the ecosystem. Members of the genera *Bacillus* and *Sphingomonas* are reported as heavy metal-resistant bacteria, and they are widely found in soils contaminated with various heavy metals (Gupta et al., 2024). Moreover, MPs particles provide attachment sites for these microorganisms. Important members of biofilms on the surface of MPs particles, *Bacillus* and *Sphingomonas* species, help break down MPs. This study also revealed that the dominant bacterial phyla in the soil microbiota were Acidobacteria and Actinobacteria. Acidobacteria, important members of the soil microbiota, play a key role in degrading plant residues and conducting photosynthesis, among other functions.

In this work, the addition of PGPR to the microbial communities in the 6.5 μm PP + Cd treatment and 830 μm PP + Cd treatment increased the relative abundance of Acidobacteria. These findings suggest that the addition of PGPR can increase the abundance of Acidobacteria in soil, increasing the degradation of plants under combined pollutant stress and thereby reducing the occurrence of disease in hybrid Pennisetum. Actinobacteria species can secrete enzymes such as cellulase, chitinase, and peroxidase, which are vital for the mineralization of organic materials in soil (Jose et al., 2021). Additionally, they can produce antibiotics that help prevent certain soil-borne diseases (Kaur et al., 2023). In this study, the relative abundance of Actinobacteria species increased to varying degrees with the addition of PGPR. Long-term inoculation with these bacteria can alter the population of Actinobacteria, thereby affecting the rate of soil material cycling and indirectly influencing the occurrence of soil-borne diseases. These findings indicate that the effects of PGPR inoculation on soil microbial communities and their functions vary under different conditions.

The alterations in microbial populations caused by the combined pollution with MPs and Cd might influence a variety of metabolic activities. Current research suggests that more accurate technologies, such as metagenomic sequencing, are crucial for understanding the genomic changes induced by MPs (Duan et al., 2024; Li et al., 2022; Sun et al., 2024). However, the application of metagenomic techniques for studying the combined pollution of MPs and Cd is relatively limited.

Through metagenomic analysis, different MPs particle sizes and the addition of different PGPR significantly altered key pathways, such as carbohydrate transport, the biosynthesis of cell wall/membrane/envelope components, and amino acid transport and metabolism, compared with those in the PP + Cd-contaminated samples. Among the KEGG level 1 pathways, metabolic pathways presented the highest abundance and absolute dominance in the hybrid Pennisetum plants. These findings indicate that the biological metabolic activity of hybrid Pennisetum is high, providing a material foundation for plant growth and development. This finding is similar to the findings of Feng et al. (2022) who reported that the addition of PE and PVC can increase the abundance of membrane transport proteins and signal transduction pathways. The functional difference analysis of the samples revealed significant variations in amino acid transport and metabolism among all the different treatment groups. Additionally, coenzyme transport and metabolism, signal transduction mechanisms, energy production and conversion, and carbohydrate transport and metabolism were key differentially abundant pathways. The above-identified functional groups were also the main components identified in other studies analyzing microbial functions in MPs-contaminated soils. These functions may play crucial roles in microbial tolerance to MPs and heavy metal pollution (Bao et al., 2022). Overall, the metabolic functions of rhizospheric soil microorganisms varied across the different treatments, and the microbial diversity also differed. These findings suggest that the stress of combined heavy metal and MPs pollution can affect microbial diversity and function. After the addition of PGPR, both metabolic functions and microbial diversity improved, indicating that these bacteria can alleviate the stress caused by the combined effects of MPs and heavy metals on plants and soil microorganisms.

## 5 Conclusion

This work examined the use of PGPR to reduce the stress resulting from combined pollution of MPs and heavy metals in hybrid Pennisetum by means of a pot experiment. The effects of the dominant PGPR strains on the bacterial community and its functions during this process were investigated. The results showed that PP particles with a size of 6.5 μm induced stronger combined stress from heavy metals on the hybrid Pennisetum plants. PGPR effectively alleviated the combined stress of the PP particles (of different sizes) and Cd, significantly increasing the length and dry weight of the hybrid Pennisetum plants. PGPR, which are microorganisms that increase plant development and remove heavy metals, can help plants absorb and accumulate less heavy metals. Metagenomics was used to examine the effects of PGPR on the composition and function of the root-associated microbiota in the hybrid Pennisetum plants. PGPR alleviated the toxicity of the combined PP + Cd contamination by altering the composition and function of the root microbiome of the hybrid Pennisetum plants. Although four strains of PGPR were used in this study, the effects of each single strain were analyzed separately through individual inoculation. The use of synthetic microbial communities is an important research direction in the biological remediation of contaminated soils. The next step should be to construct and synthesize microbial consortia using different PGPRs to increase the efficacy and stability of the PGPR. In addition, this study focused on only one MPs (PP) and one heavy metal (Cd). However, in the real world, contaminated soils are often cocontaminated with multiple types of MPs. Therefore, future research should investigate different types of MPs (e.g., polyethylene and polystyrene) and how they interact with heavy metal contamination to enhance ecological relevance.

## Data Availability

The sequences data reported in this study have been deposited in NCBI SRA with the accession number PRJNA1221826.
