# Supplementary material for: Rhizosphere microbiome metagenomics in PGPR-mediated alleviation of combined stress from polypropylene microplastics and Cd in hybrid Pennisetum
Source: Front Microbiol. 2025 Feb 17;16:1549043. doi: 10.3389/fmicb.2025.1549043 (PMC11873806; doi:10.3389/fmicb.2025.1549043)
Supplement: Supplementary file 1 [file Data_Sheet_1.PDF]

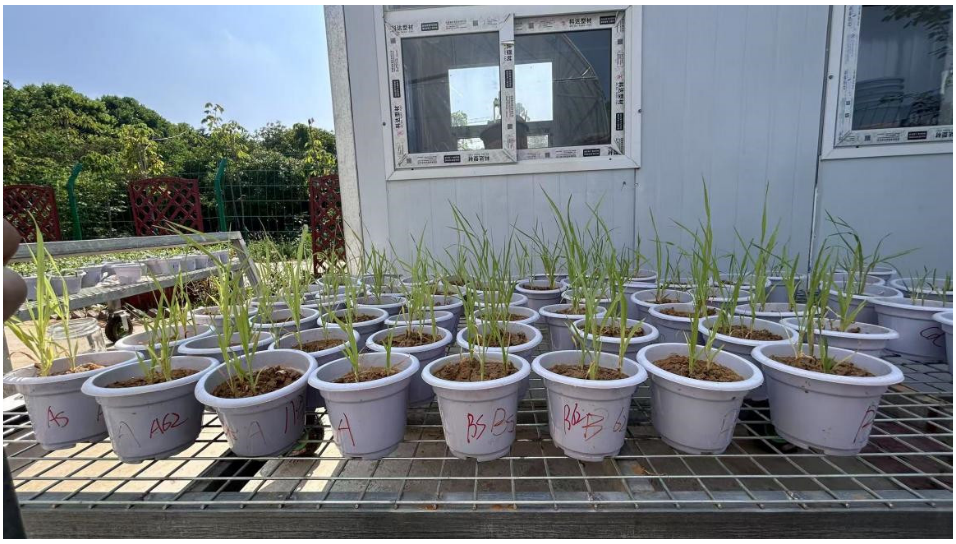

Figure S1 Photos of hybrid Pennisetum growth in pots

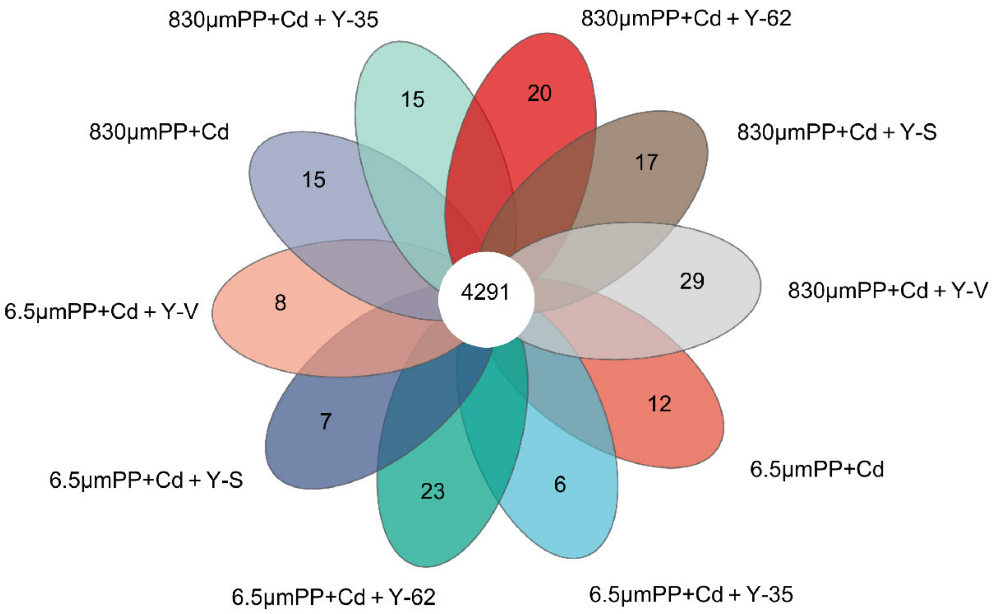

Figure S2 Venn diagram of soil microbial genera in the rhizosphere of hybrid Pennisetum under different treatments

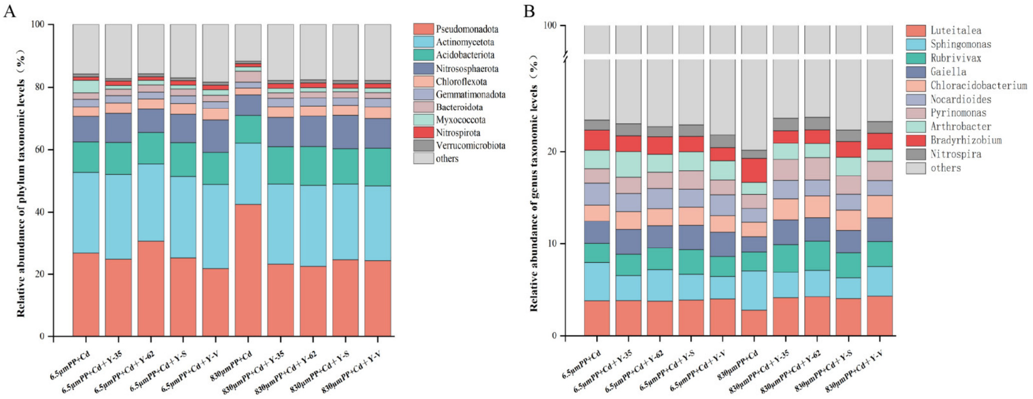

Figure S3. Relative abundance distribution of the phylum (A) and genus (B) levels community
